# Risk Factors for Recurrent Respiratory Infections in Preschool Children in China

**Published:** 2013-09-18

**Authors:** LI Hai-feng, ZOU Yan, JIN Pei-gang, JIN Hong-xing

**Affiliations:** 1 The Children’s Hospital Zhejiang University School of Medicine; 2 Zhejiang Provincial Center for Disease Control and Prevention; 3 Yiwu Women and Children’s Hospital, China

**Keywords:** Recurrent Respiratory Infection, Risk Factor, Child, Floating Population

## Abstract

***Objective:*** To identify and compare risk factors for recurrent respiratory infections in preschool children between resident and floating population in Yiwu, China.

***Methods:*** Investigations was conducted in resident and floating population in Yiwu city, Zhejiang province. A structured questionnaire was used to collect information on influence factors. Univariate and multivariate analyses were carried out using the Binary logistic regression.

***Findings*** ***:*** The study was conducted in Yiwu from June 2009 to September 2010. We recruited 844 participants，resident population 639（75.7%） and floating population 205（24.3%） who were preschool children. Multivariate analysis showed that Childbearing age (OR=0.93, 95%CI: 0.88-0.99), Asthma (OR=3.20, 95%CI: 1.60-6.45), Rickets (OR=5.30, 95%CI: 1.99-14.09), Food preference (OR=1.57, 95%CI: 1.02-2.43), Snack (OR=1.50, 95%CI: 1.07-2.07) were the influence factors for recurrent respiratory infections in resident preschool children. Infant feeding (OR=2.24, 95%CI: 1.31-3.81), Snack (OR=2.06, 95%CI: 1.07-3.98,), Trip mode (OR=2.77, 95%CI: 1.11-6.94) were the influence factors for recurrent respiratory infections in floating preschool children.

***Conclusion:*** Public health measures against risk factors should be taken to protect against recurrent respiratory infections in resident and floating preschool children respectively.

## Introduction

China has the largest floating population in the world. The floating population refers to the large and increasing number of migrants without local household registration status. Most of these migrants came from the rural areas of the central and western parts of China. They face daunting problems particularly with access to health care, adequate housing, employment opportunities. The children of floating population can not fully enjoy the social medical services, and that should bring a bad impact on their health. 

 According to a systematic analysis reported in Lancet, of the estimated 8.795 million deaths in children younger than 5 years worldwide in 2008, infectious diseases caused 68%, with the largest percentages due to pneumonia^[^^1^^]^. According to the concept of recurrent respiratory tract infections (RRIs), a child with upper respiratory infection at least 6 times or lower respiratory infection at least 2 times per year was defined as a child with recurrent respiratory infection^[^^2^^]^. Diagnosis of rhinitis, nasopharyngitis, oropharyngitis, tonsillitis or laryngitis, Otitis media was evaluated as upper respiratory tract infections. Tracheitis, bronchitis, bropnchiolitis and pneumonia were assessed as low respiratory tract infections. 

 Some literatures reported the associated factors for recurrent respiratory infections, such as socioeconomic status, family characteristics, air pollution, smoking, maternal age, breast feeding, micronutrient Condition of children et al^[^^3^^-^^10^^]^. But often only a few risk factors have been studied at a time. The present study aimed to identify and compare risk factors for recurrent respiratory infections in preschool children between resident and floating population in Yiwu, China; and to give suggestions for the health care provider to prevent recurrent respiratory infections according to the key findings for resident and floating population children respectively.

## Subjects and Methods

***Study Area and Population***

**Y**iwu, located in the central part of Zhejiang Province, covers an area of 1,105 sq km with 6 towns and 7 sub-districts and has 740,000 resident populations and 1,430,000 floating population. The study was conducted in Yiwu from June 2009 to September 2010. According to the principle of random sampling, we recruited 844 participants，resident population 639 (75.7%) and floating population 205 (24.3%) who were preschool children. They were sampling among nursery schools registered in the bureau of education in Yiwu. The sample size was calculated according the prevalence rate and the feasibility of the investigation. The sampling procedure was as follows. The registered children were coded and then selected by the method of random number which generated by Microsoft Office Excel with the function RAND. Children with congenital disease, hereditary disease and tumor as well as having a history of surgery were excluded from this study. Children using immunosuppressive agent or hormonal drugs were also excluded. In this paper, floating children were defined as preschool children who had urban registered permanent residence of Yiwu and lived in Yiwu for three and more months.

***Diagnosis procedure for RRIs***

Diagnosis was made by a physician according to their hospital records. Based on the clinical concept and management of recurrent respiratory tract infections in children of 3-5 years old in 2008^[^^2^^]^, a child with upper respiratory infection at least 6 times or lower respiratory infection at least 2 times per year was defined as a patient with recurrent respiratory infection. The interval between every two infections should be at least 7 days. If upper respiratory infections were less than 6 times annually, lower respiratory infections could be added to meet the diagnostic criteria.

***Data Collection***

In the present study, when questioning whether participants had experienced infections, diagnosis was required by a physician according to hospital records. In order to explore the influence factors in preschool children with recurrent respiratory infections, structured questionnaires were addressed the mother or guardians. The questionnaire was designed according to the refereces^[^^3^^,^^4^^]^. The first part of the questionnaire included demographic details, such as age, gander, weight and height. The second part consisted of questions about the associated factors of RRIs, including the following item categories: birth characteristics, socioeconomic factors, fostering factors,housing conditions. 

 A medical doctor and a registered nurse who was familiar with anthropometric measurements (weight, height) of children were trained for 2 days before the questionnaire investigation. The investigation was performed between June 2009 and September 2010. The serum of children was collected after questionnaire investigation. All the guardians who took part in the study gave their informed consent for participation. Trace element and Immunoglobulin in serum of the children were measured by atomic absorption method and immunological turbidity kit (Beijing Pu MB5) respectively. The laboratory results were also recorded.

***Statistical Methods***

The database was established by Epidata3.0 and analyzed by SPSS16.0 software. Chi square test was used to compare the distribution of some health related factors in preschool children between resident and floating population. If *p* value was less than 0.05, then statistical significance was considered.

 Risk factor analyses were performed in resident and floating population respectively. Risk factor analyses were carried out using Chi-square test. Based on the results of Chi-square test; a multivariate regression model was constructed by Binary logistic regression. The SPSS16.0 software was used for the Logistic Model, Forward likelihood ratio was chosen as a method of analysis. For inclusion in the model *P*=0.05 was used, whereas *P*=0.10 was chosen for exclusion from the model. In all the analyses, the confidence level was 95%.

## Findings

***Study Population and Influence Factor Analyses***

For resident and floating population respectively, the height of preschool children were 104.610.6cm and 103.69.9cm with no significant difference (*t*=1.154，*P*=0.2); the weight were 17.44.5kg and 16.7 3.7kg with significant difference (t=2.190，*P*=0.03). Childbearing age were 27.54.0 years and 26.83.9 years respectively with significant difference (*t*=0.744, *P*=0.04); the birth weight were 3.4±0.6kg and 3.3±0.6kg with no significant difference (*t*=-0.744, *P*=0.5).

 The prevalence rate for recurrent respiratory infections in resident populations and floating population children were 25.2% and 23.9% respectively with significant difference(*t*=2.190, *P*=0.03). Tables 1, 2 present the comparison of related social factors, fostering factors and living environment factors between resident populations and floating population preschool children in Yiwu, China. When the gender, parents occupation, monthly income were similar, education (father and mother) were significantly different. There were significant differences between resident populations and floating population preschool children on a number of fostering and living environment factors (delivery, vaccination, sleeping habit, taking care). 

***Risk Factors in Resident Preschool Children with Recurrent*** **Respiratory Infections**

The height of children were 104.811.0cm and 103.99.5cm in children with RRIs and control respectively, and there was no significant difference between the two groups (*t*=0.949, *P*=0.343). The weight were 17.54.6kg and 17.14.2kg in the two groups, and there was no significant difference between the two groups(*t*=0.958, *P*=0.338). 

**Table1:** Comparison of related social factors in preschool children between resident and floating population in Yiwu, China, 2009-2010

**Characteristics**		**Resident ** **population**	**Floating ** **population**	***χ***^2^	***P. *** **value**
**Gender**	Male	373 (58.4)	114 (55.6)	0.379	0.5
	Female	266 (41.6)	91 (44.4)		
**Occupation**	Brain work	324 (50.7)	96 (46.8)	1.367	0.5
	Physical force work	88 (13.8)	34 (16.6)		
	Both	227 (35.5)	75 (6.6)		
**Monthly income(RMB)**	<1000	28 (4.4)	12 (5.9)	3.255	0.4
	>=1000	170 (26.6)	46 (22.4)		
	>=3000	228 (35.7)	68 (33.2)		
	>=6000	213 (33.3)	79 (38.5)		
**Education, father**	Junior school	134 (21.0)	57 (27.8)	16.673	<0.001
	Senior school	176 (27.5)	76 (37.1)		
	College	329 (51.5)	72 (35.1)		
**Education, mother**	Junior school	139 (21.8)	82 (40.0)	32.665	<0.001
	Senior school	199 (31.1)	65 (31.7)		
	College	301 (47.1)	58 (28.3)		

**Table 2:** Comparison of related social factors in preschool children between resident and floating population in Yiwu, China, 2009-2010

**Characteristics**		**Resident ** **population**		**Floating ** **population**		***χ*** ^ 2^	***P***
**Gender**	Male	373	58.4%	114	55.6%	0.379	0.538
	Female	266	41.6%	91	44.4%		
**Occupation**	Brain work	324	50.7%	96	46.8%	1.367	0.505
	Physical force work	88	13.8%	34	16.6%		
	Both	227	35.5%	75	36.6%		
**Monthly income(RMB)**	<1000	28	4.4%	12	5.9%	3.255	0.354
	>=1000	170	26.6%	46	22.4%		
	>=3000	228	35.7%	68	33.2%		
	>=6000	213	33.3%	79	38.5%		
**Education, father**	Junior school	134	21.0%	57	27.8%	16.673	<0.001
	Senior school	176	27.5%	76	37.1%		
	College	329	51.5%	72	35.1%		
**Education, mother**	Junior school	139	21.8%	82	40.0%	32,665	<0.001
	Senior school	199	31.1%	65	31.7%		
	College	301	47.1%	58	28.3%		

Childbearing age were 27.73.9 years and 27.04.2 years in children with RRIs and control respectively, and there was significant difference between the two groups (*t*=1.969, *P*=0.049). The birth weight were 3.30.5kgand 3.40.7kg in the two groups, and there was no significant difference between the two groups (*t*=-1.459, *P*=0.145).

Tables 3, 4 present the results of the univariate risk factor analyses in resident populations preschool children. A number of factors were significantly associated with recurrent respiratory infections (delivery, asthma, rickets, food preference, snack, drinking water, sleeping habit, trip mode, someone smoking in the same room). 

 The variables were chosen as those with a *p *value of less than 0.10 in the univariate analyses. The multivariate equation model consisted of the following variables: childbearing age (OR=0.93, 95%CI: 0.88-0.99, *P*=0.01), asthma (OR=3.20, 95%CI: 1.60-6.45, *P=0.001*), rickets (OR=5.30, 95%CI: 1.99-14.09, *P=0.001*), food preference (OR=1.57, 95%CI: 1.02-2.43, *P=0.04*), snack (OR=1.50, 95%CI: 1.07-2.07, *P=0.02*). There was no significant difference between children with RRIs and control on trace element and immunoglobulin in serum (*P* >0.05) (Table 5).

**Risk Factors in Floating Preschool Children with Recurrent respiratory Infections**

The height of children were 102.79.3cm and 105.810.6cm in children with RRIs and control respectively, and there was no significant difference between the two groups (*t*=-1.945, *P*=0.05). The weight were 16.23.3kg and 17.84.2kg in the two groups, and there was significant difference between the two groups (*t*=-2.648, *P=0.009*). Childbearing age were 27.13.9years and 26.03.8 years in children with RRIs and control respectively, and there was no significant difference between the two groups (*t*=1.718, *P*=0.09). The birth weight were 3.30.6kg and 3.40.6kg in the two groups, and there was no significant difference between the two groups (*t*=-1.078, *P*=0.3).

Tables 3, 4 present the results of the univariate risk factor analyses in floating population preschool children rural child. A number of factors were significantly associated with recurrent respiratory infections (infant feeding, rickets, and snake). The variables were chosen as those with a *P* value of less than 0.10 in the univariate analyses. The multivariate equation model consisted of the following variables: infant feeding (OR=2.24, 95%CI: 1.31-3.81, *P=0.003*), snack (OR=2.06, 95%CI: 1.07-3.98, *P=0.03*), trip mode (OR=2.77, 95%CI: 1.11-6.94, *P =0.03*). 

 There were significant differences between children with RRIs and control on ferrum and alkaline phosphatase level when detecting trace element and immunoglobulin in serum. Preschool children with recurrent respiratory infection have lower level of ferrum and high level of alkaline phosphatase (*P* <0.05) (Table 5). 

**Table 3:** Risk factors for recurrent respiratory infections in preschool children between resident and floating population in Yiwu, China, 2009-2010

**Characteristics**			**Resident population**					**Floating population**			
			**Children with RRIs (%)**		**Control (%)**	***P. value***	**Children with RRIs (%)**		**Control (%)**		***P. value***
**Age(year)**	**<=3**			24 (14.9)	90 (18.8)	0.09	11 (22.4)		36 (23.7)		0.2
	**<=4**			57 (35.4)	120 (25.1)		11 (22.4)		45 (29.6)		
	**<=5**			38 (23.6)	126 (26.4)		9 (18.4)		38 (25.0)		
	**>5**			42 (26.1)	142 (29.7)		18 (36.7)		33 (21.7)		
**Gender**	**Male**			93 (57.8)	279 (58.4)	0.9	25 (51.0)		88 (57.9)		0.4
	**Female**			68 (42.2)	199 (41.6)		24 (49.0)		64 (42.1)		
**Monthly income (RMB)**	**<1000**			6 (3.7)	21 (4.4)	0.09	3 (6.1)		9 (5.9)		0.6
	**>=1000**			46 (28.6)	124 (25.9)		9 (18.4)		36 (23.7)		
	**>=3000**			46 (28.6)	186 (38.9)		15 (30.6)		54 (35.5)		
	**>=6000**			63 (39.1)	147 (30.8)		22 (44.9)		53 (34.9)		
**Delivery**	**Spontaneous labor**			78 (48.4)	190 (39.7)	0.048	29 (59.2)		84 (55.3)		0.6
	**Abdominal delivery**			83 (51.6)	290 (60.7)		20 (40.8)		68 (44.7)		
**Vaccination**	**Yes**			146 (90.7)	436 (91.2)	0.8	36 (73.5)		129 (84.9)		0.07
	**No**			15 (9.3)	42 (8.8)		13 (26.5)		23 (15.1)		
**Infant feeding**	**Exclusively breastfed**			75 (46.6)	225 (47.1)	0.9	16 (32.7)		79 (52.0)		0.03
	**Partially breastfed**			38 (23.6)	117 (24.5)		11 (22.4)		32 (21.1)		
	**Not breastfed**			48 (29.8)	136 (28.5)		22 (44.9)		41 (27.0)		
**Asthma**	**Yes**			23 (14.3)	29 (6.1)	0.001	6 (12.2)		11 (7.2)		0.3
	**No**			138 (85.7)	449 (93.9)		43 (87.8)		141 (92.8)		
**Rickets**	**Yes**			44 (27.3)	133 (27.8)	0.9	7 (14.3)		43 (28.3)		0.04
	**No**			117 (72.7)	345 (72.2)		42 (85.7)		109 (71.7)		
**Food preference**	**Yes**			92 (57.1)	225 (47.1)	0.03	25 (51.0)		60 (39.5)		0.2
	**No**			69 (42.99)%	253 (52.9)		24 (49.0)		92 (60.5)		
**Snack**	**Occasionally**			63 (39.1)	225 (47.1)	0.01	13 (26.5)		70 (46.1)		0.001
	**Regularly**			77 (47.8)	223 (46.7)		26 (53.1)		75 (49.3)		
	**Frequently**			21 (13.0)	30 (6.3)		10 (20.4)		7 (4.6)		
**Drinking water**	**Drink**			46 (28.6)	122 (25.5)	0.001	11 (22.4)		36 (23.7)		0.5
	**Plain boiled water**			97 (60.2)	338 (70.7)		33 (67.3)		108 (71.1)		
	**Fresh Fruit juice**			18 (11.2)	18 (3.8)		5 (10.2)		8 (5.3)		
**Sleeping habit**	**With adults**			120 (74.5)	313 (65.5)	0.003	27 (55.1)		84 (55.3)		0.8
	**With himself or herself**			26 (16.1)	64 (13.4)		9 (18.4)		33 (21.7)		
	**Both**			15 (9.3)	101 (21.1)		13 (26.5)		35 (23.0)		
**Trip mode**	**Car**			97 (60.2)	344 (72.0)	0.005	27 (55.1)		105 (69.1)		0.1
	**Walking**			44 (27.3)	106 (22.2)		18 (36.7)		33 (21.7)		
	**Bicycle**			20 (12.4)	28 (5.9)		4 (8.2)		14 (9.2)		
**Someone with CRD in the same room**		**Yes**		19 (11.8)	41 (8.6)	0.2	9 (18.4)		12 (7.9)		0.04
		**No**		142 (88.2)	437 (91.4)		40 (81.6)		140 (92.1)		
**Someone smoking in the same room**		**Yes**		90 (55.9)	207 (43.3)	0.006		27 (55.1)		62 (40.8)	0.1
		**No**		71 (44.1)	271 (56.7)			22 (44.9)		90 (59.2)	
**Sweeping the room frequently**		**Yes**		126 (78.3)	404 (84.5)	0.07		38 (77.6)		132 (86.8)	0.2
		**No**		35 (21.7)	74 (15.5)			11 (22.4)		20 (13.2)	

CRD: chronic respiratory disease

**Table 4:** Multivariate analysis on risk factors for recurrent respiratory infections in preschool children between resident and floating population in Yiwu, China, 2009-2010

**Characteristics**	**Resident population**											
	**Mean**	**SE**	**Wald**	***P*** ** value**	**OR**	**95% CI**	**Mean**	**SE**	**Wald**	***P*** ** value**	**OR**	**95% CI**
**Age**	0.004	0.10	0.002	0.9	1.00	0.82 - 1.22	-0.14	0.29	0.27	0.6	0.87	0.51 - 1.47
**Gender (Male)**	0.24	0.22	1.20	0.3	1.27	0.83 - 1.96	0.55	0.41	1.87	0.2	1.74	0.79 - 3.85
**Mother’s age at child’s birth**	-0.07	0.03	6.01	0.01	0.93	0.88 - 0.99						
**Infant feeding (Exclusively breastfed)**							0.80	0.27	8.77	0.003	2.24	1.31 - 3.81
**Without Asthma**	1.17	0.36	10.73	0.001	3.21	1.60 - 6.45						
**Without Rickets**	1.67	0.50	11.17	0.001	5.30	1.99 - 14.09	1.29	0.77	2.84	0.09	3.65	0.81 - 16.46
**No Food preference**	0.45	0.22	4.15	0.04	1.57	1.02 - 2.43						
**Snack (Occasionally)**	0.40	0.17	5.67	0.02	1.49	1.07 - 2.07	0.72	0.34	4.64	0.031	2.06	1.07 - 3.98
**Trip mode with car**							1.02	0.47	4.73	0.03	2.77	1.11 - 6.94

SE: Standard error; CI: Confidence interval

**Table5:** Laboratory factors for recurrent respiratory infections in preschool children between resident and floating population in Yiwu, China, 2009-2010

		**IgE**	**IgG**	**IgA**	**IgM**	**C3**	**ALP**	**Copper**	**Zinc**	**Calcium**	**Magnesium**	**Iron**	**Lead**	**Hgb**
**Resident population**	**Children with RRIs**	96.8 (146.6)	8.2 (1.5)	0.9 (0.5)	1.2 (0.2)	1.2 (0.2)	247.2 (73.0)	23.5 (4.1)	85.5 (35.6)	1.6 (0.2)	1.6 (0.5)	9.1 (3.1)	41.8 (15.4)	129.0 (7.5)
	**Control**	86.5 (109.3)	8.2 (1.8)	1.0 (0.4)	1.2 (0.2)	1.2 (0.2)	242.9 (64.9)	23.7 (2.7)	84.8 (10.3)	1.7 (0.5)	1.5 (0.2)	8.8 (0.8)	40.6 (15.1)	129.2 (12.1)
	***t***	0.788	0.146	-0.499	-0.381	-0.381	0.665	-0.743	-0.234	-1,197	1.089	0.862	0.889	-0.244
	***P *** **value**	0.431	0.884	0.618	0.703	0.703	0.506	0.458	0.815	0.232	0.276	0.389	0.374	0.807
**floating population**	**Children with RRIs**	98.7 (156.6)	8.2 (1.5)	0.9 (0.5)	1.2 (0.4)	1.1 (0.2)	243.92 (54.0)	24.2 (3.1)	85.0 (13.2)	1.6 (0.2)	1.6 (0.2)	8.7 (1.0)	40.7 (15.2)	127.9 (8,7)
	**Control**	70.9 (116.1)	8.2 (1.9)	0.9 (0.4)	1.2 (0.4)	1.1 (0.2)	224.8 (36.7)	23.2 (3.0)	101.3 (15.6)	1.8 (1.1)	1.6 (0.3)	9.2 (2.7)	42.7 (18.4)	126.7 (6.9)
	***t***	1.	-0.039	-0.857	1.041	0.230	2.308	1.966	1.709	-1.822	0.492	-2.010	0.731	0.864
	***P *** **value**	0.3	0.9	0.4	0.3	0.82	0.02	-0.05	0.09	0.07	0.6	0.046	0.5	0.4

RRI: respiratory tract infection Ig: Immunoglobulin; Alk ph: Alkaline phosphatase; Cu: Hgb; Hemoglobin

## Discussion

In the present study, child characteristics such as age, gender, birth characteristics; socioeconomic factors, fostering factors and housing conditions associated with childhood recurrent respiratory infections were studied in resident and floating population respectively. There were significant differences on parents’ education, delivery, vaccination, sleeping habit, taking care between resident and floating population. These differences may be related to the influence of social and environmental factors, health behaviors, and level of awareness concerning certain health conditions. 

 Child bearing age was associated with the recurrent respiratory infection. Consisted with the findings in Infante-Rivard C’s study^[^^11^^]^, childbearing age was a slight protective factor for recurrent respiratory infections with OR of 0.93 in resident children in this study, but probably due to Socio-economic differences and health awareness, maternal age was not found to be an influence factor in floating population.

 Nutritional factors take up an important role for recurrent respiratory infections by influencing body immune status. The global child disease burden attributable to maternal and child undernutrition has recently been quantified^ [^^12^^]^. Approximately 9 million children less than 5 years old die every year in the world. Acute lower respiratory tract infections (ALRI) account for approximately 20% of these deaths^[^^13^^,^^14^^]^. 

 Vitamin D plays an important role for human immune system^ [^^15^^]^. Vitamin D deficiency could result to abnormal metabolism of calcium and phosphor, and could be harmful to body immune functions. Vitamin D deficiency is now presumptively linked to a range of infectious inflammatory diseases throughout the life course and around the world ^[^^16^^]^. On the contrary, recurrent respiratory infections could result to the body stimulus status with nutrition storage exhausted; and with medicine for infection treatment, child had decreased appetite and limited outdoor exercises. These factors are also presumptively result to the deficient intake of vitamin D and calcium. In the present study, rickets was a risk factor for recurrent respiratory infections both in resident population with OR of 5.30. Also, we found that in floating population, preschool children with recurrent respiratory infection have high level of alkaline phosphatase; Alkaline phosphatase suggested Vitamin D deficiency. So interventions to improve child vitamin D status to improve the immune function could prevent recurrent respiratory infections and reduce the global burden of recurrent respiratory infections^[^^17^^]^. 

 Malnutrition is the underlying cause of approximately half of these fatal ALRIs. Four key nutritional risk factors for ALRI disease burden have been identified. These are macronutrient undernutrition, low birthweight, zinc deficiency and suboptimal breastfeeding^[^^18^^]^. In floating population, preschool children with recurrent respiratory infection have lower level of ferrum. This finding was probably due to socio-economic status such as family income, parent education and their awareness of nutrition et al in floating population. We also found non-breastfeeding was a risk factor for recurrent respiratory infections both in resident population with OR of 2.24. Probably due to maternal nutritional status, working and living pressure as well as education, floating population used to select non breastfeeding. A lack of exclusive breastfeeding in the first 6 months of life increases the frequency and severity of ALRI, and the risk of death from ALRIs^[^^19^^-^^21^^]^. Secondly, breastfeeding enhances the infant’s antibody responses to respiratory pathogens and influences maturation of the immune system^[^^22^^,^^23^^]^. 

 In our study, snacks tend to be a risk factor for recurrent respiratory infections with OR of 1.50 and 2.06 in resident and floating pre-children respectively probably because of the component or addictives in the snack other than protein. Food preference was found to be a risk factor for recurrent respiratory infections with OR of 1.57 only in resident children. Food preference could result to macronutrient undernutrition or overweight which depends on the preferred food. Both macronutrient undernutrition and overweight could be the key risk factors^[^^24^^]^. 

 So we should promote breastfeeding, and integrate nutrition into other aspects of well child care, and increase both public and health professional nutritional knowledge as essential components of policy development. 

 Although asthma and recurrent respiratory infections has different mechanism, asthma was reported to be a risk factor for respiratory infection in early life and the atopic or asthmatic background is a severe enough predisposing factor for the development of later recurrent respiratory infection^[^^25^^]^. Our study does find asthma associated with the risk of recurrent respiratory infections in resident preschool children, and probably because of airway hyper reactivity in asthma children. While in floating population, this association was not found, probably because of the awareness of disease and the medical counseling behavior of their parents.

 Nitrogen oxides, sulfur dioxide, carbon monoxide, particulate pollutants in the air could be important threats for respiratory system^[^^26^^-^^28^^]^. John D.Spengler found the relationship between housing characteristics and respiratory health^[^^29^^, ^^30^^]^. In the present study, trip mode was significant in multivariate analysis in floating preschool children with OR of 2.77. An explanation could be that going out by car could avoid the traffic-related pollution. But trip mode was not found to be significant in resident population probably because of the living behavior and living condition to avoid the traffic-related air pollution.

## Conclusion

childbearing age, asthma, rickets, food preference and snack were risk factors for recurrent respiratory infection in resident preschool children; infant feeding, snack, trip mode were risk factors for recurrent respiratory infection in floating preschool children. In floating population, preschool children with recurrent respiratory infection have lower level of ferrum and high level of alkaline phosphatase. These findings suggest a number of measures should be taken to reduce the effects of risk factors on children’s health. Nutrition status especially vitamin D early surveillance should be taken, and integrate nutrition should be integrate into other aspects of well child care in both resident and floating population. Breastfeeding should be promoted and nutritional knowledge should be considered as essential components of policy development, and outdoor air pollution should be avoided by improve their living conditions in floating population.
